# Prevalence and reasons for referrals to the Endodontics Specialty Clinic at the Piracicaba Dental School

**DOI:** 10.1590/1807-3107bor-2024.vol38.0008

**Published:** 2024-01-05

**Authors:** Jéssica Rodrigues RAMOS, Juliana Delatorre BRONZATO, Eloa Cristina BICEGO-PEREIRA, Adriana DE-JESUS-SOARES, Marina Angelica MARCIANO, José Flávio Affonso ALMEIDA, Caio Cezar Randi FERRAZ, Brenda Paula Figueiredo de Almeida GOMES

**Affiliations:** (a) Universidade Estadual de Campinas – Unicamp, Piracicaba Dental School, Department of Restorative Dentistry, Piracicaba, SP, Brazil.

**Keywords:** Endodontics. Electronic Health Records, Patients. Referral and Consultation, Dental Records

## Abstract

Dental referrals to the Endodontics Specialty Clinic (ESC) are routine owing to the complexity of endodontic treatments. To obtain a better prognosis for treatment, students/dentists must perceive their technical limits. This study sought to investigate the referrals of patients to the ESC from different clinics of the Piracicaba Dental School, State University of Campinas - SP, Brazil, and check: a) the demographic profile of patients and the most commonly affected tooth; b) the clinic with the largest number of referrals; c) the reasons for referrals; d) the complexity of the cases; e) and the difficulty in assessing the referred cases based on the classification provided by the American Association of Endodontists (AAE) and Souza-Filho. The study sample consisted of patients’ electronic dental referral records from February 2015 to June 2019. A total of 1,707 patients were referred to the ESC during the study period, and 62.4% were female. Lower molars were the most frequently involved teeth (34.8%), and 60.7% of the cases were referred due to the presence of root curvature. The AAE classification showed prevalence of highly difficult cases (71.3%), whereas Souza-Filho classification demonstrated a high rate of class III cases (85.8%). This study highlights the difficulties encountered by undergraduate students before or during endodontic treatments, reinforcing the need for clear criteria for selecting cases appropriate for each education level, thus improving endodontic treatment prognosis.

## Introduction

In addition to demanding basic knowledge of biology and technical fundamentals, endodontic treatment and retreatment comprise a complex clinical procedure that requires psychomotor training.^1^ Several factors may lead to procedural errors, including lack of knowledge of root canal morphology, instrumentation principles, and filling;^2^ therefore, dentists who perform endodontic procedures should be humble and ethical to assess their scientific knowledge and technical skills for a successful prognosis.^3^ In this scenario, referral to specialists is the most appropriate course of action for reducing the risk of failure in cases of greater difficulty and complexity.^4,5^

Several classifications have been developed to assess the level of difficulty of endodontic treatment and to guide dentists’ proficiency and their decision during treatment planning to refer their patients to specialists. Two classifications, however, stand out: the Endodontic Case Difficulty Assessment guideline – published by the American Association of Endodontists (AAE), and the classification proposed by Souza-Filho. The AAE^6^ describes three levels of difficulty in endodontic treatment: low, moderate, and high. Souza-Filho,^7^ on the other hand, categorizes teeth into five classes according to the level of technical difficulty and predictability.

To obtain the expected successful outcome, dental surgeons should consider the complexity of each case by evaluating canal width, root anatomy, dental anomalies, tooth position in the arch, and tooth rotation by means of radiographs.^8^ Together with digital radiographs, the health record is essential for accurate analysis and diagnosis, contributing to a better prognosis.^8-10^ Currently, all medical records, clinical files, prescriptions, and digital radiographs pertaining to the Piracicaba Dental School, University of Campinas - SP, Brazil, are stored in an electronic filing system. The electronic health record is of great importance for obtaining patient-related information and enabling an analysis of the needs and most prevalent reasons for the numerous routine referrals to specialized professionals and to different clinics within the university environment.

As part of the learning process, endodontic treatments are performed every day in the various clinics of the Piracicaba Dental School, by undergraduate, specialization, and graduate students under the direct supervision of advisors. Several cases are referred to the Endodontics Specialty Clinic for different reasons, where professionals with greater experience, knowledge of techniques, and appropriate equipment can provide more accurate treatment prognosis. After endodontic treatment, patients resume their treatments with their attending dentist.

For academic purposes and case planning, every treatment should consider the level of difficulty and the operator’s ability to perform it properly without compromising patient safety. Despite information on a large number of referrals and the reasons behind them, the actual numbers and the most common reasons for the referrals within a university environment require further investigation. Thus, this study aimed to verify the referrals of patients to the Endodontics Specialty Clinic at the Piracicaba Dental School, State University of Campinas - SP, Brazil, and to check: a) the demographic profile of patients and the most commonly affected tooth; b) the clinic with the largest number of referrals; c) reasons for referrals; d) case complexity; and e) assessment of the level of difficulty of the cases according to AAE Endodontists^6^ and Souza-Filho^7^ classifications.

## Methodology

This study was approved by the Human Research Ethics Committee of the Piracicaba Dental School (protocol number CAAE 4.047.008). Data consisted of referrals from different clinics of the Piracicaba Dental School to the Endodontics Specialty Clinic at the same institution between February 3, 2015 and June 4, 2019, collected from the electronic health and dental records.

Information on patients’ sex, self-defined color, age, and tooth elements involved were collected from the records, as well as clinics, reasons for referrals, and digital periapical radiographs. Digital periapical radiographs and tooth elements described in the referral records of each patient were evaluated to verify whether the reasons for referral specified in the dental records were compatible with what was observed on the radiographs.

The assessment of difficult was based on the AAE^6^ and Souza-Filho^7^ classifications. Briefly, in the AAE classification, low difficulty refers to uncomplicated procedures such as the treatment of wide and straight canals in anterior teeth that may lead to a predictable outcome when performed by a competent professional with limited experience. Moderate difficulty implies a complicated preoperative medical condition or the treatment of curved posterior teeth with reduced visibility, in which case a competent and experienced professional may achieve a predictable outcome. Finally, high difficulty involves an exceptionally complicated preoperative condition, including either numerous factors related to moderate difficulty or at least one factor from the high difficulty category, such as non-aligned crown and highly constricted, curved, and “C-shaped” root canals. The treatment of these cases requires a highly experienced professional with a a comprehensive track record of favorable outcomes.^6^

In the Souza-Filho classification, class I refers to wide or moderately wide, straight, or slightly curved root canals, which may be treated by undergraduate students and newly graduated professionals. Class II involves straight and/or slightly curved constricted canals, whose treatment would be ideally performed by newly graduated professionals with some clinical experience. Class III teeth include calcified canals with or without curvature, canals with pronounced root curvature, root dilacerations, canals with double curvature, presence of extra canals, endodontic retreatments, crowns with intraradicular posts, root or pulp chamber perforations, broken instruments, difficulties in achieving total isolation and mouth opening, and patients with severe systemic disorders. The endodontic treatment of these cases should be ideally performed by specialists. Class IV teeth involve dental trauma (avulsions, dislocations, and root fractures) and incomplete root development, thus requiring experienced specialists. Finally, class V includes teeth with anomalies such as dens invaginatus, fusion, gemination, and taurodontism, and such cases should also be referred to experienced specialists.^7^

Statistical analyses were performed using the Statistics Package for the Social Sciences (SPSS) for Windows (SPSS Inc., Chicago, USA). The data were tabulated and the frequencies and percentages were calculated. Associations were calculated using the chi-square test for independence. The significance level was set at 5%. When a significant association was found (p < 0.05), Cramer´s V was calculated to check the strength of that association. The values ranged from 0 to 1, where 0 indicated no association and 1 indicated a perfect association. The associations were interpreted as follows: very weak for 0.0 to <0.01; weak for 0.1 to < 0.2; moderate for 0.2 to < 0.4; and strong for ≥ 0.4.^11^

## Results

Between February 3, 2015 and June 4, 2019, there were 1,707 referrals from different clinics of the Piracicaba Dental School to the Endodontics Specialty Clinic. Most of the patients were white (80.1%), female (62.4%), and older than 30 years (81.5%) (Table 1). The most affected teeth were lower molars (34.8%), followed by upper molars (29.1%) (Table 1).

**Table 1:** Clinical features of patients referred from different clinics of the Piracicaba Dental School to the Endodontics Specialty Clinic.

Variable	n	%
Sex		
Female	1,066	62.4
Male	641	37.6
Ethnicity/color		
White	1,367	80.1
Black	138	8.1
Brown	195	11.4
Yellow	7	0.4
Age (years)		
≤ 30	315	18.5
> 30	1,392	81.5
Tooth location		
Upper arch	926	54.2
Lower arch	781	45.8
Tooth element		
Upper central incisor	110	6.4
Upper lateral incisor	94	5.5
Upper canine	43	2.5
Upper premolar	182	10.7
Upper molar	497	29.1
Lower central incisor	15	0.9
Lower lateral incisor	29	1.7
Lower canine	26	1.5
Lower premolar	117	6.9
Lower molar	594	34.8

The undergraduate outpatient clinic accounted for the largest number of referrals (72.2%), followed by the emergency department (15.3%) (Table 2). Endodontic treatment (62.4%) and endodontic non-surgical retreatment (29%) were the most prevalent reasons for referral (Table 3), but case evaluation, surgical treatment, and trauma were also accounted for as reasons for the referrals.

**Table 2:** Number and percentage of referrals from different clinics of the Piracicaba Dental School to its Endodontics Specialty Clinic.

Clinic	n	%
Undergraduate outpatient clinic	1,233	72.2
Emergency department	262	15.3
Prosthodontics specialty clinic	67	4
Pathology specialty clinic	54	3.2
Periodontics specialty clinic	51	3
Restorative dentistry specialty clinic	40	2.3

**Table 3:** Number and percentage of the reasons for referrals and complexity of the cases referred from different clinics of the Piracicaba Dental School to its Endodontics Specialty Clinic.

Variable	n	%
Reason		
Endodontic treatment	1,065	62.4
Endodontic non-surgical retreatment	495	29
Evaluation	132	7.7
Trauma treatment/follow-up	10	0.6
Endodontic surgery	5	0.3
Complexity		
Root curvature	1,037	60.7
Root atresia	293	17.2
Removal of cast metal post/cores	57	3.3
Difficulty in rubber dam isolation	49	2.9
Removal of glass fiber post	45	2.6
Root canal deviation	41	2.4
Broken instruments	28	1.6
Root canal perforation	27	1.6
Patients with special needs	26	1.5
Glass fiber post fracture	6	0.4
Cast metal post/core fracture	3	0.2
Dental anomalies	2	0.1
Not specified in the records	93	5.5

Root canal curvature (60.7%) and root canal atresia (17.2%) were the most common complexities encountered by the attending dentists (Table 3).

Most of the referred cases were classified as presenting high difficulty (85.8%) according to the AAE classification, followed by moderate (18.7%) and low difficulty (2.2%) (Table 4). When the Souza-Filho classification was applied, most cases were classified as class III (85.7%) (Table 4). Cases referred for evaluation (7.7%) were not assessed as to their level of difficulty. Tables 1, 2, 3, and 4 show these findings in more detail.

**Table 4:** Assessment of the level of difficulty of the cases referred from different clinics of the Piracicaba Dental School to its Endodontics Specialty Clinic.

Level of difficulty classification	n	%
AAE (2019)		
Low	38	2.2
Moderate	320	18.7
High	1,217	71.3
Souza-Filho (2015)		
Class I	37	2.2
Class II	62	3.6
Class III	1,464	85.8
Class IV	10	0.6
Class V	2	0.1

The results showed a moderate association between the tooth element and AAE and Souza-Filho classifications. Also, there was a moderate association between the clinic where the referral was made and the reason for referral (Table 5).

**Table 5:** Cramer’s V correlation coefficient of the patients’ characteristics.

Variable	Reason	Clinic	AAE Classification	Souza-Filho Classification	Number of reasons
Sex	0.076*	0.088*	-	-	0.085*
Age	0.096**	0.15***	0.077*	0.081*	-
Dental arch	0.104**	-	-	0.091*	-
Tooth element	0.183***	0.113***	0.226***	0.257***	0.182***
Self-defined color	0.082***	0.093***	0.07*	-	-
Clinic	0.245***	NA	0.177***	0.107***	0.141***

*p < 0.05, **p < 0.01, ***p < 0.001, (-) not significant for chi-square test for independence. NA, not applicable.

## Discussion

This study analyzed the electronic dental records and radiographs of patients from the Piracicaba Dental School to investigate the reasons underlying patients’ referral from different clinics of the university to the Endodontics Specialty Clinic. We also sought to verify the most commonly affected dental group, sex, age, color/ethnicity, degree of complexity of the case to be referred, and the classification of the level of difficulty.

Most referrals were for female patients. These findings corroborate those reported in the literature, indicating that most dental patients are women.^12,13^ This may be due to the fact that females, in general, act more positively toward oral health and dental visit than do males.^14^ Although routine exams tend to detect asymptomatic pulpal and periapical diseases, as well as the need for treatment, our study did not investigate endodontic diagnoses prior to the referrals.

In our study, most patients identified themselves as white (80.1%), whereas only 8.1% identified themselves as black, 11.4% as brown, and 0.4% as yellow – with brown indicating white and black ancestors and yellow representing Asian. Considering that 47.73% of the Brazilian population consists of white individuals, 43.13% brown, 7.61% black, 1.09% yellow, and 0.43% indigenous,^15^ the rates indicate a racial disparity in dental care. However, such disparity cannot be attributable to purchasing power because endodontic treatment at the Piracicaba Dental School is free of charge; therefore, cultural differences may be involved.^16,17^

Our results show that molars accounted for the tooth element with the greatest treatment difficulty, 594 (34.8%) of which were related to the lower molars – corroborating the findings reported in the literature.^18,19^ As it is the first permanent tooth to erupt in the oral cavity and given its complex anatomy, the permanent mandibular first molar is more susceptible to caries.^12,20^ Thus, lower molars are more susceptible to pulpal and periapical diseases. Upper molars were the second most widely affected teeth (29.1%). Molars have more complex anatomy than that of other teeth, with usually three or four root canals, and two or three roots. They are also located in the posterior region of the mouth, which hinders endodontic treatment.^21^

The undergraduate outpatient clinic accounted for most of the referrals to the Endodontics Specialty Clinic (72.2%), a finding that reflects the need for training qualified professionals to treat more complicated cases,^22^ allowing them to gain confidence in their treatment skills.^23^ Conventional endodontic treatments, retreatment, treatment of traumatized teeth, and the need for endodontic surgery were described as reasons for referral to the Endodontics Specialty Clinic, corroborating the reports of another study.^24^ We also found 132 (7.7%) cases referred for evaluation of the real need for endodontic treatment/retreatment. These were probably borderline cases where the decision to keep the teeth in the oral cavity would depend on their restorability and absence of advanced periodontal disease.

There was a moderate association between the clinic where the referral was made and the reason for referral. This reflects the need of each clinic, for example, undergraduates receive patients mostly for treatment and when they refer them is because they encounter complexities that they are not able to treat.

Among the complexities found for endodontic treatments/retreatments, root curvature was the most prevalent reason for referral (60.7%). This finding contradicts those reported in different studies, which found broken instruments and calcified root canals as the main reasons for referral.^24,25^ However, these studies investigated the reasons for referrals by general dentists in private practices, not within the university environment.

We also found the presence of atresic canals, removal of glass fiber post, removal of cast metal post/cores, difficulties in rubber dam isolation, root canal deviations, perforations, broken instruments, and dental anomalies as complexities associated with endodontic treatments. Patients with special care needs such as those presenting with pregnancy, HIV, uncontrolled heart rate, non-Hodgkin’s lymphoma, and those on alendronates were also described as reasons for referral. These cases demonstrate the concern of dental students or even specialists from other areas with treatment that is beyond their skills and knowledge, resulting in the referral to professionals with better qualifications. This finding corroborates those reported in other studies.^24,26, 27^

By looking at the digital radiographs obtained from the electronic record system, we found that most referred cases were classified as high difficulty (71.3%), according to the AAE. Such difficulties are very similar to those described in the Souza-Filho classification, where most cases were categorized as class III (85.8%). This explains the association between tooth elements and the classifications, as most difficulties classified as class III or high difficulty are encountered in molars (the most frequently involved teeth).

This study highlights the difficulties encountered by undergraduate students before or during endodontic treatment/retreatment, reinforcing the need for criteria that allow referring cases according to educational level within the Endodontics course. Treatment planning also functions as an important tool for screening at and referral to the undergraduate outpatient clinic, helping improve specialization and graduate programs in the field.

The results indicate the need for an inclusive public health program to raise awareness of the importance of dental care, follow-up, and check-up visits among individuals of all ethnic groups and genders. Dental students should acknowledge the importance of orienting patients in their first dental visit and reinforcing the information during further visits. Students should learn how to treat a patient as a whole individual rather than to focus solely on dental care. They should, therefore, learn how to treat patients with systemic disorders, which will then help students build their confidence in the provision of healthcare.

The treatment of teeth with pronounced root curvatures and root atresia requires specialization training. Undergraduate students should be trained to develop their skills related to diagnosing and referring patients to specialists, thus improving patients’ prognosis. Students should therefore graduate with not only the necessary information and skills to perform proper endodontic treatments, but also with the ability to refer patients who are beyond their treatment capacity, understanding the need to seek further education to provide their patients with better treatment. Future research could include the analysis and comparison of referral systems within other universities.

## Conclusion

This study highlighted the difficulties encountered mainly by undergraduate students before or during endodontic treatments, reinforcing the need for criteria for the referral of cases according to educational level within endodontics programs, enabling the improvement of endodontic treatment prognosis.
